# An Intensive Lifestyle Intervention Is an Effective Treatment of Morbid Obesity: The TRAMOMTANA Study—A Two-Year Randomized Controlled Clinical Trial

**DOI:** 10.1155/2015/194696

**Published:** 2015-07-15

**Authors:** Bartolomé Burguera, Juan Jesús Tur, Antonio Jorge Escudero, María Alos, Alberto Pagán, Baltasar Cortés, Xavier Francesc González, Joan B. Soriano

**Affiliations:** ^1^Instituto Universitario de Investigación en Ciencias de la Salud (IUNICS), Hospital Universitario Son Espases, Carretera de Valldemossa 79, 07010 Palma de Mallorca, Spain; ^2^Endocrinology and Bariatric Institutes, Cleveland Clinic, 9500 Euclid Avenue M62, Cleveland, OH 44195, USA; ^3^Unidad de Investigación, Hospital Universitario Son Espases, Palma de Mallorca, Spain; ^4^Cirugía General, Hospital Universitario Son Espases, Palma de Mallorca, Spain; ^5^Instituto de Investigación Hospital Universitario de la Princesa (IISP), Universidad Autónoma de Madrid, Cátedra UAM-Linde, 28030 Madrid, Spain

## Abstract

Bariatric surgery is currently the most effective therapy to induce weight loss in morbidly obese patients. *Objective*. This controlled, clinical trial with a two-year intervention was aimed at comparing the efficacy of two nonsurgical approaches versus bariatric surgery, on body weight changes and metabolic parameters in morbidly obese patients. *Methods*. Patients were randomized to an Intensive Lifestyle Intervention (ILI) (*n* = 60) or Conventional Obesity Therapy (COT) (*n* = 46). The ILI group received behavioral therapy and nutritional counseling. The COT group received standard medical treatment. They were compared with a third group, Surgical Obesity Group (SOG) (*n* = 37). *Results*. Patients who received ILI had a greater percentage of weight loss than patients receiving COT (−11.3% versus −1.6%; *p* < 0.0044). Interestingly 31.4% of patients included in the ILI group were no longer morbidly obese after just six months of intervention, increasing to 44.4% after 24 months of intervention. The percentage weight loss in SOG was −29.6% after that same period of time. *Conclusions*. ILI was associated with significant weight loss when compared to COT, in a group of patients with obesity. An ILI approach could be an alternative therapy to patients with obesity, who are not candidates to undergo bariatric surgery. This trial is registered with EudraCT 2009-013737-24.

## 1. Introduction

Obesity has become an epidemic of global proportions. Morbid obesity (MO) [body-mass index (BMI) > 40 Kg/m^2^] is considered the most serious stage of the disease, with a population prevalence of 6–8% in the US [1] and 1-2% in Spain [2]. Therefore, many people are living with a higher risk of dying from complications of their obese status, or living with a disability [3] and/or a psychosocial stigma [4, 5] that prevents them from enjoying a satisfying life. The general recommendation to morbidly obese patients, to lose 10% of their initial body weight [6, 7], is well known to have a positive impact reducing their cardiovascular risk.

Nowadays we can offer two therapeutic approaches to our patients with MO: the Conventional Obesity Therapy (COT) currently provided in our endocrine/obesity clinics, or bariatric surgery. Unfortunately, it is becoming evident that COT has very limited effect, as the Swedish Obesity Study [8] and other studies have shown [9–11]. Bariatric surgery, however, is currently the most effective treatment that we can offer to the large majority of our patients with MO [8, 12–15].

The Socialized Health System in Spain provides weight loss surgery to less than 6,000 patients with MO every year [16]. The bariatric surgery waiting list in public hospitals averages more than two years in Spain and other European Union countries, and it is estimated that, with the current strategy, only one in every 200 patients with MO will undergo bariatric surgery [17]. There is indeed a significant mismatch, between patients who potentially could benefit from a bariatric procedure and the actual number of subjects who undergo surgery. The associated costs of taking care of these morbidly obese patients, many of them with important comorbidities, are therefore expected to increase in the near future. Patients with MO, in many instances, are visited by different specialists due to their multiple comorbidities, while no one addresses comprehensively their main medical problem, which is their obesity.

This problem is acquiring dramatic proportions and underlines the need to implement and develop efficacious obesity treatments (alternative to bariatric surgery), so that we can effectively treat our nonsurgical candidates, morbidly obese patients. There are already some studies showing the beneficial effects of behavioral +/− pharmacotherapy interventions [18–23]. However, few of these studies have included patients with MO in the context of a prospective, randomized clinical trial.

We designed a 24-month controlled, clinical trial with a six-month follow-up, to compare the health impact of an Intensive Lifestyle Intervention (ILI) on patients with MO, compared to Conventional Obesity Therapy (COT) and bariatric surgery. We aimed to test whether an ILI was effective in helping patients with MO to significantly lose weight and maintain this weight loss six months after the trial.

## 2. Materials and Methods

### 2.1. Participants

This report expands our previous trial publication from one year to two years plus a six-month follow-up period [24]. Individuals with MO (BMI > 40 Kg/m^2^) were selected from our obesity clinic in Son Espases University Hospital in Mallorca (Spain). These patients were not paid to participate, nor did they pay any medical costs. All patients provided written informed consent to participate in the study. Additional written informed consent was obtained prior to any surgical procedure. The study was reviewed and approved by the Balearic Islands Human Ethics Committee (reference number IB 451/05) in accordance with the guidelines of the Helsinki Declaration.

Inclusion criteria were (1) age between 18 and 65 years, (2) men or women of any ethnic group with a BMI > 40 Kg/m^2^, (3) arterial pressure < 160/100 mmHg, (4) a fasting triglycerides concentration < 600 mg/dL, and a glycosylated hemoglobin (HbA1c) level < 11%. The exclusion criteria included drug or alcohol abuse, pregnancy, enrollment in other obesity interventions, previous bariatric surgery, mental disorders (depression and anxiety which were considered manageable by the principal investigator were not criteria for exclusion) and/or physical impairment, or any other criteria which could interfere with the ability to comply with treatment as previously detailed [24].

### 2.2. Intensive Lifestyle Intervention (ILI) Group

Patients assigned to receive ILI behavioral modification attended weekly group meetings from week one through week 12. Subsequently, sessions were conducted biweekly from week 13 to week 52. Meetings lasted for 90 minutes, included 10 to 12 subjects, and were led by a registered nurse with a master's in nutrition. The group sessions were focused on the qualitative aspects of dietary habits, as the distribution of energy intake, frequency of consumption, and food choices. We provided information on the benefits of the Mediterranean diet and encouraged our patients to comply with this diet. There were no restrictions in caloric intake. Subjects were instructed to complete weekly homework assignments during a 72-hour period to develop perspectives on food preferences and meal patterns.

A sports medicine specialist prescribed daily exercise (led by a physiotherapist), physical self-checks, and stretching without resistance, in sets of two minutes every four to six hours coordinated with breath control, before performing aerobic exercise.

Patients, who were eligible, could receive treatment with weight loss medicines, such as Orlistat (Xenical, Roche, USA), or antidepressants at the physician's discretion. Forty per cent of the patients included in this group received treatment with sibutramine (Meridia, Abbot Laboratories, USA) for a period of only one to two months until it was withdrawn from the market in January of 2010.

### 2.3. Conventional Obesity Therapy (COT) Group

Patients in this group received standard available nutritional education, medical treatment, and follow-up available for MO patients as per the Spanish Endocrine Society protocol. Patients had regular clinic visits with an endocrinologist, dietitian, and nurse every three to six months throughout the duration of the study. Medical therapies, including the use of pharmacological agents, were determined by their endocrinologist on an individual basis. Only 15% of patients received weight loss medications.

### 2.4. Surgical Obesity Group (SOG)

The SOG was constituted by those patients, already included in the Bariatric Surgery Program at our hospital and it was intended to be an additional control group. We included 37 consecutive patients who were willing to participate. Patients underwent a laparoscopic biliopancreatic diversion (BPD) which is routinely performed at our Institution, following guidelines of the Spanish Obesity Society (SEEDO) [25].

### 2.5. Objectives

The main objective of the TRAMOMTANA study (*Tratamiento Multidisciplinar de la Obesidad Mórbida: Medicamentos, Terapia de comportamiento, Apoyo Nutricional y Actividad Física*, or Multidisciplinary Treatment of Morbid Obesity: Drugs, Behavioral Therapy, Nutritional Support and Physical Activity) was to evaluate, in patients with morbid obesity, the impact of an intensive, multidisciplinary, nonsurgical weight loss program, including nutritional assessment, promotion of physical activity, medications, and support in the process of change in lifestyle.

### 2.6. Primary and Secondary Outcomes

The primary outcomes of body weight (Kg) and BMI (Kg/m^2^) were measured at all study visits. Height and weight were measured with the subjects barefooted and lightly dressed, with a Tanita's WB-3000 digital beam scale with a mechanical height rod, allowing height to be measured the same time as weight in the first visit. Height was taken at head level to the nearest centimeter, with the subject standing barefooted, with feet together and following the plane of Frankfurt. BMI was calculated as weight in kilograms divided by the square of height in meters. Circumferences were measured to the nearest cm using a flexible tape with the subject standing. Weight was measured weekly during the first three months, biweekly for 21 months in the ILI, and every three months in the COT and bariatric surgery groups, during routine visits, with both surgeon and endocrinologist.

Secondary outcome measures included percent changes in fasting plasma glucose (FPG), glycosylated hemoglobin (HbA1c), and blood pressure (BP) and of fasting lipids obtained at baseline and every three months after initiation of the intervention. A 40 mL fasting blood sample was obtained from an antecubital vein. Samples were prepared using low speed centrifugation at 4,000 g for ten minutes and stored in our hospital Registered Biobank. Major obesity-related comorbidities, including hypertension, hypercholesterolemia, and hypertriglyceridemia, were assessed for changes after intervention by evaluation of blood tests. Seated BP and pulse were measured on each occasion in duplicate, using an automated device after a five min rest.

### 2.7. Randomization-Sequence Generation

**Figure 1:**
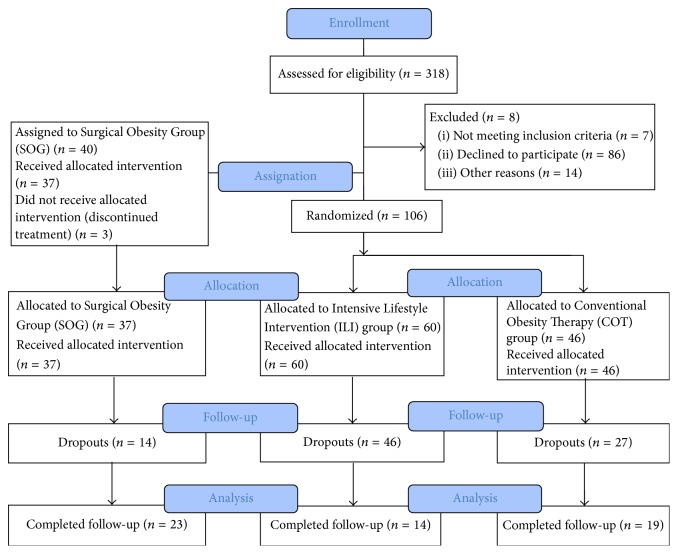
Consort flow chart follow-up 24 + 6.

Randomization to either ILI or COT was computer-derived, after all eligibility criteria were confirmed, and the study was not blinded (Figure 1).

### 2.8. Sample Size and Statistical Analysis

The sample size was calculated with enough power to be able to detect clinically significant effects in percent changes of weight from baseline and one year after intervention (*p* > 0.80). Given the longitudinal character of the study and the need for changes in food patterns, we expected that 50% of the patients in the intervention group (ILI) would not finish the trial, because of losses to follow-up, so we conservatively increased the sample size to 60 subjects randomized to the intervention arm of the study (ILI group), that is, 50% of the sample size. The first control group (COT group) would hold a proportion of 25% of the sample size; and finally the SOG group would hold the remaining proportion of 25%. Given a type one error of 5%, with 80% power, and taking into account a baseline BMI of 45 Kg/m^2^, with a standard deviation of 4-5 Kg/m^2^, and considering a clinically relevant reduction of BMI to be at least four units in kg/m^2^, equivalent to a 10%, we required a minimum sample size of 60 ILI, 30 COT, and 30 SOG patients.

We compared normalized continuous variables between groups at baseline using analysis of variance and Fisher's test. We compared categorical variables at baseline using chi-square test. Changes at the 24th month for continuous variables were expressed as overall percentage unitary variation (mean ± SD; percentage unitary change between initial value and end value; paired samples, i.e., percentage change per unit patient) in comparison with the same patients at baseline. It was tested by a planned sequential procedure in which an analysis of variance was performed first and Bonferroni pairwise analyses were performed to compare treatments in case of global significance.

Continuous variables are reported as mean ± SD and categorical variables as number and percentage. A *p* value less than 0.05 was considered statistically significant. Data were analyzed using R-project version 2.12.0.

## 3. Results

The baseline demographic and clinical characteristics of the three study groups are shown in Table 1. The mean age of participants was 46.5 ± 10.7 years, 31.4% were male, and 98.3% were Caucasian. There were no racial/ethnic differences, body weight, or other parameters at baseline among the three groups. Just the BMI of the SOG group was higher, due to a weight bias selection in the patient included in the surgical waiting list.

### 3.1. Weight Loss

After two years of intervention, patients who received ILI had a higher percentage of weight loss than patients in the COT group, −11.3 ± 8.7 (ILI), −1.6 ± 7 (COT) % weight Loss (Table 2).

**Figure 2:**
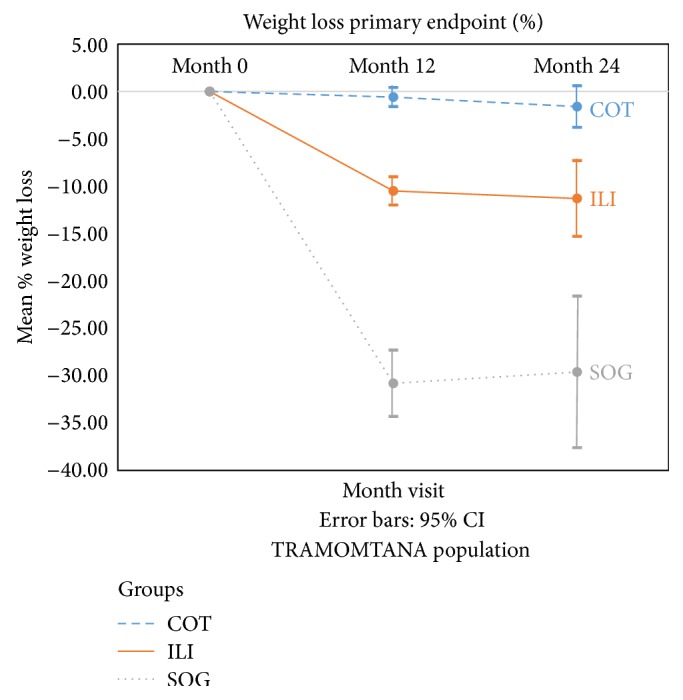
Percent weight loss from baseline, one year, two years.

Figure 2 shows that % weight loss (primary endpoint), in the COT, ILI, and SOG groups, respectively, was 0.6%, −10.5%, and −30.8% for year one and −1.6%, −11.3%, and −29.6% for year two; and finally at the end of the study the % weight loss was −3.3%, −9.6%, and −30.3%.

Figure 2 also depicts BMI (secondary endpoint), in the COT, ILI, and SOG groups: 47.2 ± 5.3, 45.8 ± 5.5, and 49.5 ± 5.7, respectively, at baseline; 46.37 ± 4.83, 40.15 ± 5.33, and 33.83 ± 5.82, at the end of year one; 46.2 ± 5.4, 40.6 ± 6.9, and 34.4 ± 6.1 at the end of year two; and finally, after the follow-up period, the BMI was 46.4 ± 5.1, 39.6 ± 5.1,  and  34.1 ± 6.4, respectively (Table 3).

**Figure 3:**
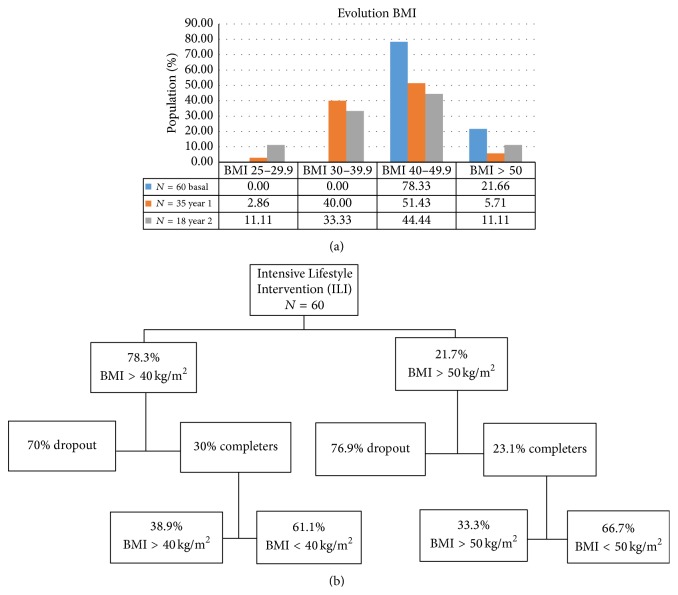
Changes in BMI up to year two, by morbid obesity thresholds.

As expected, the weight loss achieved with ILI was variable (Figure 3(a)) and we identified four levels of weight loss response, after two years of intervention. 21.6% of patients in the ILI group were super obese (BMI > 50 Kg/m^2^) and 78.3% had a BMI >40–<50 kg/m^2^. Of interest, 61% of patients who completed the two years of ILI therapy had accomplished a reduction of BMI to non-MO levels, and therefore they were not candidates for bariatric surgery anymore. Also 11.1% of ILI patients achieved a BMI < 30 kg/m^2^ that would classify them as only overweight patients. The percentage of super obese patients (BMI > 50 kg/m^2^) who completed the two-year study was 23.1%. However, 66.7% of these patients had their obesity category from super obese to MO (Figure 3(b)).

21.7% of the subjects in the COT group discontinued the study at year one, and this number increased to 36.9% at the end of year two (Table 2). 41.7% in the ILI group did not complete the one-year study, whereas 70% of participants had dropped the study after two years (Table 2). This number included anyone who attended the first treatment visit but did not return. Seven subjects (18.9%) in the surgical group were lost to follow-up within the first year and 37.8% did not show up for their 24-month follow-up visit. The patients who abandoned the study did so because of changes in social and employment status or the development of medical complications unrelated to the intervention that prevented them from complying with the study protocol.

### 3.2. Impact on Biochemical Markers of Type 2 Diabetes

Mean FPG in mg/dL decreased after one year of ILI, from 135.6 ± 45.2 to 112.3 ± 37.3 mg/dL, compared to a change from 119.3 ± 47.3 to 124 ± 63.2 mg/dL in the COT group as previously shown [25]. Data at two years showed that the mean FPG improved by 12.7%  ±  24.2 in the ILI group while the COT group increased 4.3%  ±  30.8%. However, these differences were not statistically significant (*p* = 0.09). The SOG's mean FPG decreased from 106.8 ± 20.5 to 92.1 ± 14.5 mg/dL.

We also compared HbA1c changes in the subgroup of Type 2 diabetic patients included in the three arms of the study. While there were no statistically significant differences among the three groups, HbA1c slightly improved in all COT patients (−3.1%  ±  20.5%) and in the SOG (−8.4%  ±  9.8%), while patients in ILI did not change (0.4%  ±  9%) (Table 2).

### 3.3. Impact on Other Biomarkers

In lipid levels, HDL-C levels showed an improvement in all three groups (also both in men and in women) after two years of participation in the trial, except for women included in the COT group. Regarding TG levels, both ILI and SOG participants showed reductions in their % change plasma levels, 8.7 ± 27.2 and 15.7 ± 32.7 mg/dL, respectively, compared to no changes in those receiving COT (−0.1 ± 21.6 mg/dL). However, these differences were not statistically significant among the three groups at two years (Table 2).

Mean LDL-C level did not show statistically significant improvement in the ILI group compared to the COT group after two years, whereas patients from the SOG showed a significant improvement in this parameter (*p* < 0.0001). The mean level of LDL-C in the SOG showed an improvement (27.7%  ±  21.4%), which was statistically significant compared to the other two groups; ILI 5.2%  ±  22.2% and COT −1.5%  ±  16.4% (*p* < 0.0001).

Finally, there was a significant reduction of serum triglyceride levels in the ILI group of −8.7 ± 27.2% after two years of intervention compared with a small change in triglyceride levels (−0.1 ± 21.6%) in the COT group. The surgical group also showed an improvement in their triglyceride levels (−15.7 ± 32.7%) (Table 2).

### 3.4. Blood Pressure and Heart Rate

Systolic and diastolic blood pressure levels were neither significantly different among the three groups, nor significantly different after one year or two years of intervention. Pulse showed a significant improvement in the ILI and COT compared to SOG, after two years of intervention; however baseline pulse values were originally higher at baseline in these two medically treated groups. There were no statically significant differences in pulse values among the three groups after two years of intervention (Table 2).

### 3.5. Surgical Outcomes and Complications

Thirty-seven patients underwent bariatric operations (biliopancreatic diversion). The median length of hospital stay was eight days. The overall major complication rate was 2%. Among the most frequent ones were infection 3%, bleeding 2%, gastrointestinal fistula 2%, and pulmonary thromboembolism 0.2%. Minor complication rate was 10%. These included gastrointestinal (diarrhea 15%), nutritional deficiencies (iron 25%, Vitamin B12 30%, calcium/Vitamin D 50%, and hypoalbuminemia 5%), incisional hernias (3%), pneumonia (2%), and wound infections (5%).

### 3.6. Six-Month Extension after Trial Completion

We asked all patients who had completed the two-year intervention to come back for a follow-up visit six months after the completion of the study. Fourteen subjects in the ILI group attended this 30-month follow-up visit, whereas 19 patients in COT and 23 in SOG came to this visit.

At this follow-up evaluation, six months after the end of the intervention period, patients who received ILI continued to have a higher percentage of weight loss as compared to patients in COT. Namely, subjects in ILI maintained a weight loss of −9.6%  ±  7.8% Kg compared to the weight loss of −3.3%  ±  7.3% in the COT group (*p* = 0.23) (Table 3). The surgical group maintained a weight loss of 30.3%  ±  12.6%.

The mean fasting plasma glucose continued to be better in the ILI group (−16.4%  ±  22.4%) compared to the COT group (−1%  ±  28.1%). The SOG group showed a fasting glucose improvement of 11%  ±  18.4%, but these differences were not statistically significant (*p* = 0.1517). The HbA1c was slightly lower in the type 2 diabetic patients included in SOG compared to the ILI and COT patients, but again there were no statistically significant differences among the three groups (Table 3) (*p* = 0.1). LDL-C was improved in the SOG group, which was statistically significant when compared to the other two medical groups (*p* < 0.05) (Table 3). Neither systolic nor diastolic blood pressure nor heart rate changed significantly among the three groups after this 6-month follow-up period.

## 4. Discussion

Previous studies have shown the benefits of ILI compared to COT in obese patients [18–23]. The results of this two-year clinical trial TRAMOMTANA extend these observations to patients with MO and compare them to patients undergoing bariatric surgery. The morbidly obese patient population is a subgroup of obese population whose relative size is increasing the fastest and consumes the largest proportion of the Health National Budget (due to its comorbidities), and approximately less than 2% of MO patients will eventually undergo bariatric surgery.

Current guidelines indicate that obese patients should visit a specialist in order to lose weight and prevent obesity-related conditions [26]. The reality is that a myriad of patients with MO are followed up on a regular basis (every four to six months) in our obesity clinics worldwide, with losing significant weight. Unfortunately the current medical therapeutic approach to this chronic condition is not effective in helping these patients to lose weight in the long term, at least for more than two years. Therefore, it seems imperative for the sake of our patients and our Health System that we design effective obesity therapeutic approaches in the hospital and/or primary care settings, more efficacious than our current obesity treatments and potentially an alternative therapy to patients not interested in/suitable for undergoing bariatric surgery.

Recent publications have shown the efficacy of ILI on obese and type 2 diabetic patients with MO [9, 11, 20]. In the Look AHEAD study at year one, participants in the ILI had lost 8.6 kg of initial body weight, compared to 0.7 kg only in the COT group. At year 4, participants in the ILI maintained a weight loss of 4.9 kg in comparison with only 1.3 kg loss, in COT. In a two-year randomized clinical trial, Ryan et al. [11] showed that patients with MO randomized to ILI in a primary care setting lost a significant amount of weight, compared to those receiving COT (21% of patients lost 10% or more of the initial weight). In agreement with the data presented in this manuscript, the authors reported a weight loss of 5% or higher in 31% of patients and a 10% weight loss in 21% of cases, with a significant improvement in several metabolic parameters. They also reported that retention (51% in the ILI group) and weight loss maintenance were two key points which had to be improved in subsequent studies.

There is general agreement that one of the major challenges of all weight loss programs is to maintain any weight loss achieved, over the medium and long term period [25]. It is important that patients undertake lifestyle changes durable enough, to allow a significant improvement in their comorbidities, quality of life [27, 28], and body composition [29].

In a one-year nonrandomized controlled trial [30], Johnson et al. showed that an ILI intervention was associated with more favorable dietary changes than gastric bypass surgery in MO patients, as measured by intake of vegetables, whole grains, dietary fiber, and saturated fat. Others suggest that current weight loss programs usually achieve a reduction of 7 to 10% of the initial body weight [31, 32] after six to nine months of intervention, and the combination of diet, physical activity, and behavioral changes can obtain even better results if antiobesity drugs are added [33].

TRAMOMTANA is, to our knowledge, the first randomized trial comparing two medical treatments (ILI versus COT) to induce weight loss in patients with MO, with a two-year duration. Results presented in this paper indicate that MO patients included in an ILI obtained an average weight loss (10%) significantly better compared to the group of patients receiving COT (1-2%). Patients who followed our supervised ILI program were more prone to incorporate healthy lifestyles and exercise in their daily routine. This study also expands the findings of previous reports, showing the benefits of lifestyle modifications, by inducing clinically significant weight loss in nonmorbid obese patients [34].

Limitations of our study include the lack of randomization to the surgical arm; patients were only randomized to the two medical treatments. We could not randomize patients to bariatric surgery because our surgeons had to follow the waiting list of patients with MO in our hospital, already accepted for a bariatric procedure, and also due to ethical committee concerns. The limited sample size of this study, however, reached significance in most major and minor aims, and the fact that it was open label might limit the strength of the recommendations. As expected, the dropout rate was significant, however, in agreement with previous clinical trials involving patients with MO [11]. It is important to emphasize that during the recruitment process patients were informed of the possibility of receiving sibutramine therapy, if they were candidates. The fact that this medication was removed from the market two months after patients had started therapy and they had to stop treatment was a major contributing factor to our significant dropout rate. Our study was not powered for safety or to detect differences in endpoints such as mortality or cardiovascular events. Despite these limitations, TRAMOMTANA strongly supports that ILI represents a potentially effective weight loss strategy for management of patients with MO. This effect should significantly improve, if weight loss medications are added to ILI. It is important to point out that ILI patients significantly lost weight after receiving advice on a more healthy lifestyle and Mediterranean diet, while there were no restrictions in caloric intake and they did not use dietary supplements or low caloric diets.

We consider that our findings are relevant, given the evident mismatch between the number of patients with MO and the actual number of MO patients who may undergo a bariatric procedure. Despite the much larger weight loss observed in the surgical group, an average body weight loss of 10% obtained with ILI represents a reduction of four units of BMI, which can produce an important health benefit for our patients, a 40% reduction in our bariatric surgery waiting list, and important health costs savings.

Finally, our trial also underlines an important issue which is currently receiving little notoriety. It is the fact that the current therapeutic approach to treat obesity in general and MO patients in particular, in our clinics, is not efficacious and perhaps not cost-effective. Our results provide strong support for the recommendation that ILI can be used as therapy of MO in the obesity clinic and the incorporation of ILI therapy in obesity therapy programs should be seriously considered. The addition of weight loss medications should be seriously considered. ILI programs can help to better identify which patients are suitable for benefitting from medical therapy, without the need of being included in the bariatric surgery waiting list. Programs like TRAMOMTANA should be considered in an effort to early identify patients, who could benefit from these interventions, so we can maximize resources and refer to bariatric surgery only the patients who have previously failed a lifestyle program, instead of patients who have just failed to comply with the limited advice received in biannual medical visits. Furthermore many insurance companies require, following a medically supervised weight loss program, before giving approval to obese patients, undergoing a bariatric procedure.

Likely, the addition to ILI programs of the recently approved new weight loss medications should make these ILI interventions even more effective. Until we can help our patients with MO to lose weight and to keep it off with a medical weight loss program, bariatric surgery will continue to be the most efficacious way to treat only a minority of our patients with MO.

To conclude, the TRAMOMTANA two-year trial provides strong evidence to support the recommendation of ILI interventions to induce significant weight loss for the treatment of morbidly obese patients. ILI (with or without combination with antiobesity drugs) could be their last chance to regain control over their weight (and their health), especially if bariatric surgery is not a feasible option.

## Figures and Tables

**Table 1:** Demographic and clinical characteristics of patients^*∗*^.

Characteristic	ILI (*N* = 60) total population	COT (*N* = 46) total population	SOG (*N* = 37) total population	*p* value^†^
Age, yr	47.8 ± 11.5	46.9 ± 10.3	44.1 ± 9.8	0.2533
Male/female sex, number (% male)	17/43 (28.3)	17/29 (37.0)	11/26 (29.7)	0.6164
Weight, Kg	122.2 ± 20.1	126 ± 17.9	132.8 ± 24.4	0.0526
Height, cm	1.63 ± 0.1	1.64 ± 0.1	1.64 ± 0.1	0.8161
Body-mass index^‡^	45.8 ± 5	46.8 ± 4.6	49.2 ± 5.9	0.0068
Systolic blood pressure	131.9 ± 18.7	136.1 ± 14	132.6 ± 14.4	0.3933
Diastolic blood pressure	86.8 ± 10	86.6 ± 10	82.8 ± 8.9	0.1129
Heart rate	82.9 ± 11.7	89 ± 10.6	81 ± 7.9	0.0014
Cholesterol (mg/dL)				
Total	202.8 ± 37.2	194.4 ± 31.8	178.8 ± 38	0.0067
High-density lipoprotein	49.4 ± 10.4	44.1 ± 9.1	40.2 ± 6.2	<0.0001
Low-density lipoprotein	121.8 ± 31.8	119.2 ± 23	118 ± 28.9	0.7915
Triglycerides (mg/dL)	162 ± 62.4	151.4 ± 66.7	136.5 ± 63.9	0.1647
Fasting glucose (mg/dL)	122.1 ± 40.1	116.4 ± 40.6	110.4 ± 26.9	0.3242
Glycated hemoglobin, %	6.7 ± 1.7 (*N* = 15)	7.8 ± 1.7 (*N* = 11)	6.4 ± 0.8 (*N* = 9)	0.2291
Diabetes, number (%)	15 (25)	11 (23.9)	9 (24.3)	0.9905
Study level				
Basic, number (%)	42 (70.0)	33 (71.7)	34 (91.89)	0.0333
Medium, number (%)	12 (20.0)	9 (19.6)	3 (8.1)	0.2601
High, number (%)	6 (10.0)	4 (8.7)	0 (0.0)	0.148
Marital status				
Single, number (%)	11 (18.3)	12 (26.1)	11 (29.7)	0.3985
Married, number (%)	37 (61.7)	26 (56.5)	15 (40.5)	0.1208
Widowed, number (%)	4 (6.7)	2 (4.3)	2 (5.4)	0.8744
Separated & divorced, number (%)	8 (13.33)	6 (13.03)	9 (24.32)	0.2847
Birth country				
Spain, number (%)	58 (96.7)	42 (91.3)	34 (91.9)	0.4611
Latin American, number (%)	1 (1.7)	3 (6.5)	2 (5.4)	0.4256
Rest of Europe, number (%)	1 (1.7)	1 (2.2)	1 (2.7)	0.9411
Tobacco use				
Current smoker, number (%)	9 (15.0)	10 (21.7)	7 (18.9)	0.6659
0–20, number (%)	1 (11.1)	7 (70.0)	5 (71.4)	0.0307
>20, number (%)	8 (88.9)	3 (30.0)	2 (28.6)	0.3993
Past or never, number (%)	51 (85.0)	36 (78.3)	30 (81.1)	0.6659
Race				
Caucasian, number (%)	59 (98.3)	44 (95.6)	35 (94.6)	0.5788
Other races, number (%)	1 (1.7)	2 (4.3)	2 (5.4)	0.5788
Diabetes, number (%)	15 (25.0)	11 (23.9)	9 (24.3)	0.9905
Fasting glucose (mg/dL)	122.1 ± 40.1	116.4 ± 40.6	110.4 ± 26.9	0.3242
Glycated hemoglobin, %	6.7 ± 1.7 (*N* = 15)	7.8 ± 1.7 (*N* = 11)	6.4 ± 0.8 (*N* = 9)	0.2291

^*∗*^Plus-minus values are means ± SD.

^†^ *p* values are for comparisons within the three groups.

^‡^The body-mass index is the weight in kilograms divided by the square of the height in meters.

ILI: Intensive Lifestyle Intervention, COT: Conventional Obesity Therapy, and SOG: Surgical Obesity Group.

**Table 2:** Changes at 24 months.

Variable	ILI (*N* = 18)		COT (*N* = 29)		SOG (*N* = 23)		*p* value overall	*p* value^*∗*^		
								ILI versus COT	ILI versus SOG	COT versus SOG
Weight loss^†^	123.3 ± 27.2	109.5 ± 26.7	127.1 ± 18.3	125.1 ± 20.2	133.6 ± 27	93.2 ± 22.7	<0.0001	0.0044	<0.0001	<0.0001
	−11.3 ± 8.7		−1.6 ± 7		−29.6 ± 13.1					

Body-mass index^†^	45.8 ± 5.5	40.6 ± 6.9	47.2 ± 5.3	46.2 ± 5.4	49.5 ± 7	34.4 ± 6.1	<0.0001	0.0041	<0.0001	<0.0001
	−11.6 ± 8.7		−1.8 ± 7		−29.7 ± 13.1					

Systolic blood pressure^†^	126.6 ± 20.3	129.4 ± 15.7	137.8 ± 12.7	138 ± 21	128.7 ± 13.9	130.4 ± 18.1	0.698	1	1	1
	4.2 ± 17.6		0.5 ± 14.9		1.6 ± 12.3					

Diastolic blood pressure^†^	84.6 ± 12.9	84 ± 12.3	86.4 ± 11.1	84 ± 11.9	78 ± 7.5	77 ± 9.6	0.7969	1	1	1
	0.4 ± 14		−2.2 ± 11.7		−0.6 ± 14.3					

Heart rate^†^	80.3 ± 11.8	70.6 ± 12	90.5 ± 12.8	80.8 ± 12.8	77.2 ± 6.4	78.1 ± 10.6	0.002	1	0.0065	0.0064
	−11.6 ± 11.8		−10.1 ± 11.8		1.9 ± 16.2					

Glucose^†^	135.6 ± 45.2	112.3 ± 37.3	119.3 ± 47.3	124 ± 63.2	106.8 ± 20.5	92.1 ± 14.5	0.0366	0.09	1	0.091
	−12.7 ± 24.2		4.3 ± 30.8		−11.5 ± 18.1					

Glycated hemoglobin^†^	6.4 ± 0.9 (*N* = 8)	6.5 ± 1.2 (*N* = 8)	7.7 ± 1.8 (*N* = 10)	7.5 ± 2.5 (*N* = 10)	6.1 ± 0.5 (*N* = 5)	5.6 ± 0.7 (*N* = 5)	0.6124	1	0.98	1
	0.4 ± 9		−3.1 ± 20.5		−8.4 ± 9.8					

Total cholesterol^†^	209.4 ± 40.6	207.9 ± 32	193.8 ± 24.5	188.8 ± 27.5	177.3 ± 37.3	149.8 ± 29.3	0.0061	1	0.011	0.027
	0.9 ± 15.3		−2.1 ± 11.1		−13.5 ± 18.9					

High-density lipoprotein^†^	49.6 ± 11.3	52.6 ± 11.3	44.1 ± 10.2	44.2 ± 9.7	40.5 ± 7.8	43.5 ± 13.3	0.5572	1	1	0.92
	6.9 ± 12.1		2.2 ± 19.4		8.3 ± 28.2					

High-density lipoprotein men^†^	41.8 ± 11.2 (*N* = 5)	43.8 ± 11.9 (*N* = 5)	35.4 ± 8.5 (*N* = 8)	37.8 ± 6.2 (*N* = 8)	34.8 ± 7.4 (*N* = 8)	38.1 ± 13.4 (*N* = 8)	0.9113	1	1	1
	5.2 ± 10.5		11.7 ± 31.8		−11.9 ± 34.9					

High-density lipoprotein women^†^	52.5 ± 10.3 (*N* = 13)	56 ± 9.5 (*N* = 13)	47.5 ± 8.9 (*N* = 21)	46.7 ± 9.8 (*N* = 21)	43.5 ± 6.2 (*N* = 15)	46.3 ± 12.8 (*N* = 15)	0.2366	0.41	1	0.54
	7.6 ± 13		−1.4 ± 11.1		6.4 ± 25					

Low-density lipoprotein^†^	126.7 ± 31	130.2 ± 30.3	120.7 ± 19.9	117.9 ± 24.1	115.8 ± 27.3	81.7 ± 24.1	<0.0001	0.79	<0.0001	<0.0001
	5.2 ± 22.2		−1.5 ± 16.4		−27.7 ± 21.4					

Triglycerides^†^	177.6 ± 73.8	150.6 ± 46	142.4 ± 49.7	138.1 ± 42.6	141.5 ± 71.1	114.7 ± 66.2	0.1235	0.88	1	0.13
	−8.7 ± 27.2		−0.1 ± 21.6		−15.7 ± 32.7					

^*∗*^ *p* values for the overall comparisons were calculated with the use of analysis of variance. *p* values for the comparisons between each pair of groups were calculated with the use of the Bonferroni method in post hoc analyses. *p* values were normalized; data were obtained from each patient percentage unitary variation (change between initial time and end time); it must be noted that this transformation does unitary variation positioning the patient on the same output level in each of the patients.

ILI: Intensive Lifestyle Intervention, COT: Conventional Obesity Therapy, and SOG: Surgical Obesity Group.

^†^The first row indicates the mean and ± standard deviation baseline measurement, and the mean and ± standard deviation measurement at two years in international units. The second row indicates the mean percentage change (%) ± standard deviation (%) by calculation according to unit paired patient from baseline to two years.

International units: weight Kg, body-mass index Kg/m^2^, systolic blood pressure & diastolic blood pressure mm/Hg, heart rate bpm, glucose & total cholesterol & high-density lipoprotein & low-density lipoprotein & triglycerides mg/dL, and glycated hemoglobin %.

**Table 3:** Changes in outcome variables at the 30th month.

Variable	ILI (*N* = 14)		COT (*N* = 19)		SOG (*N* = 23)		*p* value overall	*p* value^*∗*^		
								ILI versus COT	ILI versus SOG	COT versus SOG
Weight loss^†^	114.5 ± 19.9	103.7 ± 20.8	129.6 ± 18	125.1 ± 19	133.5 ± 27	92.5 ± 23.6	<0.0001	0.23	<0.0001	<0.0001
	−9.6 ± 7.8		−3.3 ± 7.3		−30.3 ± 12.6					

Body-mass index^†^	43.9 ± 4.2	39.6 ± 5.1	47.8 ± 5.2	46.4 ± 5.1	49.5 ± 7	34.1 ± 6.4	<0.0001	0.15	<0.0001	<0.0001
	−9.9 ± 7.8		−2.5 ± 9.2		−30.5 ± 12.6					

Systolic blood pressure^†^	125.9 ± 22.6	126.1 ± 17.5	139.2 ± 15.5	134.4 ± 18.1	128.7 ± 13.9	129.3 ± 16	0.5377	0.98	1	1
	2.2 ± 16.8		−2.9 ± 12.9		1.3 ± 14.5					

Diastolic blood pressure^†^	83.1 ± 13.8	84.2 ± 10.2	84.6 ± 12.3	81.6 ± 10.7	78 ± 7.5	73.8 ± 11.6	0.4656	1	0.66	1
	3.1 ± 16.6		−2.1 ± 17.2		−4.3 ± 18.7					

Heart rate^†^	78.07 ± 7.7	72.36 ± 5.73	88.95 ± 12.59	82.84 ± 14.45	77.22 ± 6.36	79.3 ± 6.51	0.0079	1	0.039	0.017
	−6.5 ± 12.1		−6.7 ± 9.6		3.3 ± 12					

Glucose^†^	135 ± 50.8	104.2 ± 22.5	126.4 ± 56.4	118.8 ± 43.1	106.8 ± 20.5	92.4 ± 12.9	0.1517	0.19	1	0.5
	−16.4 ± 22.4		−1 ± 28.1		−11 ± 18.4					

Glycated hemoglobin^†^	6.2 ± 0.9 (*N* = 6)	6.6 ± 0.8 (*N* = 6)	7.7 ± 1.9 (*N* = 9)	6.9 ± 1.9 (*N* = 9)	6.1 ± 0.5 (*N* = 5)	5.7 ± 0.8 (*N* = 5)	0.0965	0.12	0.32	1
	7.47 ± 6.02		−9.41 ± 18.8		−7.36 ± 11.1					

Total cholesterol^†^	212.4 ± 45.4	210.9 ± 34.2	187.1 ± 24.3	178.3 ± 25.8	177.3 ± 37.3	145.5 ± 32.7	0.0179	1	0.022	0.142
	1.6 ± 17		−4.1 ± 11.7		−15.5 ± 22.5					

High-density lipoprotein^†^	50.4 ± 12.5	51.8 ± 13.6	44.7 ± 11.9	45 ± 10.1	40.5 ± 7.8	44.4 ± 14.1	0.5462	1	1	1
	3.25 ± 12.67		3.56 ± 17.77		10.4 ± 30.78					

High-density lipoprotein men^†^	39.8 ± 11.8 (*N* = 4)	37.8 ± 7.7 (*N* = 4)	33.4 ± 10.3 (*N* = 5)	37 ± 9 (*N* = 5)	34.8 ± 7.4 (*N* = 8)	34.9 ± 8.3 (*N* = 8)	0.5637	0.93	1	1
	−2.5 ± 14.2		14.5 ± 22.2		3.5 ± 28.2					

High-density lipoprotein women^†^	54.6 ± 10.4 (*N* = 10)	57.4 ± 11.2 (*N* = 1)	48.7 ± 9.8 (*N* = 14)	47.9 ± 9 (*N* = 14)	43.5 ± 6.2 (*N* = 15)	49.4 ± 14.1 (*N* = 15)	0.2477	1	1	0.3
	5.55 ± 12		−0.34 ± 14.96		14.09 ± 32.42					

Low-density lipoprotein^†^	128.6 ± 35.2	137.7 ± 34.5	115.7 ± 21.2	108.7 ± 20.8	115.8 ± 27.3	79.2 ± 25.6	<0.0001	0.1342	<0.0001	0.0006
	10.2 ± 23.3		−4.6 ± 15.2		−29.9 ± 22.3					

Triglycerides^†^	168.8 ± 66.2	155.6 ± 55.6	135.6 ± 44.6	124.5 ± 38.4	141.5 ± 71.1	103.5 ± 39.4	0.098	1	0.2	0.21
	−3.4 ± 30.9		−5.1 ± 23.6		−20.9 ± 28.8					

^*∗*^ *p* values for overall comparisons within groups were calculated with the use of analysis of variance. *p* values for the comparisons between each pair of groups were calculated with the use of the Bonferroni method in post hoc analyses. *p* values were normalized; data were obtained from each patient percentage unitary variation (change between initial time and end time); it must be noted that this transformation does unitary variation positioning the patient on the same output level in each of the patients.

ILI: Intensive Lifestyle Intervention, COT: Conventional Obesity Therapy, and SOG: Surgical Obesity Group.

^†^The first row indicates the mean and ± standard deviation baseline measurement and the mean and ± standard deviation measurement at follow-up in international units. The second row indicates the mean percentage change (%) ± standard deviation (%) by calculation according to unit paired patient from baseline to follow-up.

International units: weight loss Kg, body-mass index Kg/m^2^, systolic blood pressure & diastolic blood pressure mm/Hg, heart rate bpm, glucose & total cholesterol & high-density lipoprotein & low-density lipoprotein & triglycerides mg/dL, and glycated hemoglobin %.

## References

[1] http://www.cdc.gov/nchs/data/hestat/obesity_adult_09_10/obesity_adult_09_10.htm.

[2] Gutiérrez-Fisac J. L., Guallar-Castillón P., León-Muñoz L. M., Graciani A., Banegas J. R., Rodríguez-Artalejo F. (2012). Prevalence of general and abdominal obesity in the adult population of Spain, 2008–2010: the ENRICA study. *Obesity Reviews*.

[3] Gregg E. W., Guralnik J. M. (2007). Is disability obesity's price of longevity?. *Journal of the American Medical Association*.

[4] Hayden M. J., Dixon M. E., Dixon J. B., Playfair J., O'Brien P. E. (2010). Perceived discrimination and stigmatisation against severely obese women: age and weight loss make a difference. *Obesity Facts*.

[5] Chen E. Y., Bocchieri-Ricciardi L. E., Munoz D. (2007). Depressed mood in class III obesity predicted by weight-related stigma. *Obesity Surgery*.

[6] World Health Organization (1998). *Obesity: Preventing and Managing the Global Epidemic*.

[7] National Institutes of Health/National Heart (1998). Clinical guidelines on the identification, evaluation, and treatment of overweight and obesity in adults. *Obesity Research*.

[8] Sjöström L., Lindroos A.-K., Peltonen M. (2004). Lifestyle, diabetes, and cardiovascular risk factors 10 years after bariatric surgery. *The New England Journal of Medicine*.

[9] Wadden T. A., Webb V. L., Moran C. H., Bailer B. A. (2012). Lifestyle modification for obesity: new developments in diet, physical activity, and behavior therapy. *Circulation*.

[10] Espeland M., Pi-Sunyer X., Blackburn G. (2007). Reduction in weight and cardiovascular disease risk factors in individuals with type 2 diabetes: one-year results of the look AHEAD trial. *Diabetes Care*.

[11] Ryan D. H., Johnson W. D., Myers V. H. (2010). Nonsurgical weight loss for extreme obesity in primary care settings: results of the Louisiana Obese Subjects Study. *Archives of Internal Medicine*.

[12] Buchwald H., Avidor Y., Braunwald E. (2004). Bariatric surgery: a systematic review and meta-analysis. *The Journal of the American Medical Association*.

[13] Scopinaro N., Marinari G. M., Camerini G. B., Papadia F. S., Adami G. F. (2005). Specific effects of biliopancreatic diversion on the major components of metabolic syndrome: a long-term follow-up study. *Diabetes Care*.

[14] Pories W. J., Caro J. F., Flickinger E. G., Meelheim H. D., Swanson M. S. (1987). The control of diabetes mellitus (NIDDM) in the morbidly obese with the Greenville Gastric Bypass. *Annals of Surgery*.

[15] Schauer P. R., Burguera B., Ikramuddin S. (2003). Effect of laparoscopic Roux-en Y gastric bypass on type 2 diabetes mellitus. *Annals of Surgery*.

[16] Lecube A., Monereo S. (2011). RICIBA (computerized registry of bariatric surgery), what do we know about bariatric surgery in Spain?. *Endocrinología y Nutrición*.

[17] Anselmino M., Bammer T., Fernández Cebrián J. M., Daoud F., Romagnoli G., Torres A. (2009). Cost-effectiveness and budget impact of obesity surgery in patients with type 2 diabetes in three European countries(II). *Obesity Surgery*.

[18] Wing R. R., Bray G. A., Bouchard C. (2004). Behavioral approaches to the treatment of obesity. *Handbook of Obesity: Clinical Applications*.

[19] Diabetes Prevention Program (DPP) Research Group (2002). The diabetes prevention program (DPP): description of lifestyle intervention. *Diabetes Care*.

[20] Wadden T. A., Neiberg R. H., Wing R. R. (2011). Four-year weight losses in the look AHEAD study: factors associated with long-term success. *Obesity*.

[21] van Baak M. A., van Mil E., Astrup A. V. (2003). Leisure-time activity is an important determinant of long-term weight maintenance after weight loss in the sibutramine trial on obesity reduction and maintenance (STORM trial). *The American Journal of Clinical Nutrition*.

[22] Goodpaster B. H., DeLany J. P., Otto A. D. (2010). Effects of diet and physical activity interventions on weight loss and cardiometabolic risk factors in severely obese adults: a randomized trial. *Journal of the American Medical Association*.

[23] Arterburn D. E., Crane P. K., Veenstra D. L. (2004). The efficacy and safety of sibutramine for weight loss: a systematic review. *Archives of Internal Medicine*.

[24] Tur J. J., Escudero A. J., Alos M. M. (2013). One year weight loss in the TRAMOMTANA study. A randomized controlled trial. *Clinical Endocrinology*.

[25] Rubio M. A., Martínez C., Vidal O. (2004). Documento de consenso sobre cirugía bariátrica. *Revista Española de Obesidad*.

[26] Mechanick J. I., Youdim A., Jones D. B. (2013). Clinical practice guidelines for the perioperative nutritional, metabolic, and nonsurgical support of the bariatric surgery patient—2013 update: cosponsored by American Association of Clinical Endocrinologists, The Obesity Society, and American Society for Metabolic & Bariatric Surgery. American Association of Clinical Endocrinologists; Obesity Society; American Society for Metabolic & Bariatric Surgery. *Obesity*.

[27] Søvik T. T., Aasheim E. T., Taha O. (2011). Weight loss, cardiovascular risk factors, and quality of life after gastric bypass and duodenal switch: a randomized trial. *Annals of Internal Medicine*.

[28] Wyatt S. B., Winters K. P., Dubbert P. M. (2006). Overweight and obesity: prevalence, consequences, and causes of a growing public health problem. *The American Journal of the Medical Sciences*.

[29] Johannsen D. L., Knuth N. D., Huizenga R., Rood J. C., Ravussin E., Hall K. D. (2012). Metabolic slowing with massive weight loss despite preservation of fat-free mass. *Journal of Clinical Endocrinology and Metabolism*.

[30] Johnson L. K., Andersen L. F., Hofso D. (2013). Dietary changes in obese patients undergoing gastric bypass or lifestyle intervention: a clinical trial. *British Journal of Nutrition*.

[31] Jeffery R. W., Drewnowski A., Epstein L. H. (2000). Long-term maintenance of weight loss: current status. *Health Psychology*.

[32] Knowler W. C., Barrett-Connor E., Fowler S. E. (2002). Reduction in the incidence of type 2 diabetes with lifestyle intervention or metformin. *The New England Journal of Medicine*.

[33] Wadden T. A., Berkowitz R. I., Sarwer D. B., Prus-Wisniewski R., Steinberg C. (2001). Benefits of lifestyle modification in the pharmacologic treatment of obesity: a randomized trial. *Archives of Internal Medicine*.

[34] Martins C., Strømmen M., Stavne O. A., Nossum R., Mårvik R., Kulseng B. (2011). Bariatric surgery versus lifestyle interventions for morbid obesity—changes in body weight, risk factors and comorbidities at 1 year. *Obesity Surgery*.

